# Proteinase-Activated Receptor 1 (PAR1) Regulates Leukemic Stem Cell Functions

**DOI:** 10.1371/journal.pone.0094993

**Published:** 2014-04-16

**Authors:** Nicole Bäumer, Annika Krause, Gabriele Köhler, Stephanie Lettermann, Georg Evers, Antje Hascher, Sebastian Bäumer, Wolfgang E. Berdel, Carsten Müller-Tidow, Lara Tickenbrock

**Affiliations:** 1 Department of Medicine, Hematology/Oncology, University of Muenster, Muenster, Germany; 2 Gerhard Domagk Institute for Pathology, University of Muenster, Muenster, Germany; 3 Interdisciplinary Center for Clinical Research IZKF, University of Muenster, Muenster, Germany; 4 Dept. of Medicine IV, Hematology and Oncology, University of Halle, Halle, Germany; 5 Hochschule Hamm-Lippstadt, University of Applied Science, Hamm, Germany; RWTH Aachen University Medical School, Germany

## Abstract

External signals that are mediated by specific receptors determine stem cell fate. The thrombin receptor PAR1 plays an important role in haemostasis, thrombosis and vascular biology, but also in tumor biology and angiogenesis. Its expression and function in hematopoietic stem cells is largely unknown. Here, we analyzed expression and function of PAR1 in primary hematopoietic cells and their leukemic counterparts. AML patients' blast cells expressed much lower levels of PAR1 mRNA and protein than CD34^+^ progenitor cells. Constitutive *Par1*-deficiency in adult mice did not affect engraftment or stem cell potential of hematopoietic cells. To model an AML with *Par1*-deficiency, we retrovirally introduced the oncogene MLL-AF9 in wild type and *Par1^−/−^* hematopoietic progenitor cells. *Par1*-deficiency did not alter initial leukemia development. However, the loss of *Par1* enhanced leukemic stem cell function *in vitro* and *in vivo*. Re-expression of PAR1 in *Par1^−/−^* leukemic stem cells delayed leukemogenesis *in vivo*. These data indicate that Par1 contributes to leukemic stem cell maintenance.

## Introduction

The four Proteinase-Activated Receptors (PAR1 to PAR4) belong to a superfamily of seven transmembrane, G-protein coupled cell-surface receptors [1]. PARs receive various extracellular signals and mediate them to intracellular responses and play a prominent role in a variety of physiological processes [2], [3]. Activation of PARs occurs usually via proteolytic cleavage of their N-terminal exodomain through extracellular proteases like thrombin. Cleavage creates a new N-terminus that serves as tethered ligand and allows the activation of intracellular signal cascades [4], [5].

PAR1 as the prototype of this group is a high-affinity thrombin receptor and it is therefore critical e.g. in thrombosis 3,6, inflammation [7], [8], [9] and angiogenesis [10]. PAR1 can also be activated by MMP-1, a matrix metalloprotease [2], [11]. Absence of Par1 is partially incompatible with embryonic development, since at least half of *Par1*-deficient mice die around embryonic day E9.5 due to severe bleeding that could be rescued by the introduction of Par1 expression in embryonic endothelial cells [10]. The surviving mice do not exhibit obvious abnormalities [12], [13]. Yue *et al*. recently demonstrated that Par1 plays a role in the *in vitro* differentiation of mouse embryonic stem cells into hematopoietic progenitors and in endothelial-to-hematopoietic transition in zebrafish [14]. However, the function of Par1 in adult hematopoiesis has not yet been addressed.

High PAR1 expression was found in tumors including malignant melanoma [15] and breast cancer [16], [17] and correlated with invasiveness and motility of numerous cancer cell lines [18], [19], [20], [21], indicating that PAR1 might act as an oncogene. Since the function of PAR1 in leukemia is yet unknown, we here present the first report about PAR1 in adult hematopoiesis and leukemogenesis. In particular, we identify PAR1 as a novel regulator of leukemic stem cells in AML in an *in vivo* mouse model.

## Materials and Methods

### Patient samples and ethics statement

The study was reviewed and approved by the ethics committee of the medical association and the medical faculty of the University of Muenster (2007-524-f-S and 2007-390-f-S) before the study began. AML samples were obtained from bone marrow of patients with acute myeloid leukemia at the time of initial diagnosis. The median blast count was 80%. For microarray analysis and RT-PCR, CD34^+^ cells were obtained from the peripheral blood of healthy donors who were stimulated with G-CSF using standard protocols. Informed written consent was obtained from all patients.

### Microarray analysis and data from the Leukemia Gene Atlas

Published microarray data from human bone marrow and blood cells were analyzed using the Leukemia Gene Atlas at http://www.leukemia-gene-atlas.org (accessed 2014 Mar 25) [22], [23]. The analyzed cells were obtained from human umbilical cord blood or from peripheral blood samples [23].

For comparison of control and AML patient samples, the mRNA of 5 healthy CD34^+^ progenitor specimens and 67 AML patient samples was hybridized on Whole Genome Microarrays. Microarray data and the patient cohort were analyzed previously [24]. Informed consent was obtained from all patients and donors.

### RNA isolation and real-time quantitative RT-PCR

RNA isolation from patient samples and murine cells was performed using RNeasy Micro Kit (Qiagen, Hilden, Germany) according to the manufacturer's protocol.

Reverse transcription and real-time quantitative RT-PCR were performed as described [25]. The probes were labeled at the 5' end with the fluorescent dye FAM (PAR1) or VIC (GAPDH) and at the 3' end with the quencher TAMRA. Primer/Probe sets were obtained from Life Technologies (Darmstadt, Germany; “Mm00438851_m1 F2r” for murine and “Hs00169258_m1 F2R” for human samples).

### Flow cytometry, mice, colony assays, limiting dilution transplantation, and competitive transplantations

FACS analyses of blood were performed as described [26]. HSC FACS and sorting for HSC subpopulations was performed as described [27].

Par1-Knockout (−/−) mice were obtained from Jackson laboratory (Stock Number: 002862) [12] and genotyped as published. Par1^−/−^ mice survived with a lower frequency than expectable according to Mendelian ratio, since we obtained only 32 Par1^−/−^ mice out of 269 pubs (12% instead of expected 25%) from matings of heterozygous parents.

All animal experiments in this study were carried out in strict accordance with the recommendations of the Institutional Animal Care and Use Committee “Landesamt fuer Natur, Umwelt und Verbraucherschutz NRW”. This study was performed with permission of the Institutional Animal Care and Use Committee and of the local veterinary administration of Muenster (Permit Numbers: G15/2005, 8.87-51.04.20.09.322, and 8.87-51.04.2011.A005).

For colony formation assays, bone marrow cells from three age-matched *Par1*-wild type and – knockout mice were flushed from femur and tibia of both hind legs using PBS/2% FCS and the red cells were lysed by AKC shock as described [26]. 10,000 cells from the total unsorted bone marrow or from c-kit^+^ bone marrow cells and sorted by FACS as described above were seeded in M3434 methylcellulose (StemCell Technologies, Inc.) and counted after 7–8 days. Replating was performed by resolving the colonies in PBS, seeding again 10,000 cells per ml methylcellulose and counting as above.

For limiting dilution analysis, limiting amounts of donor cells (100, 1000 or 10000 total bone marrow cells) from 3 pairs of *Par1^+/+^* (total of n = 44) vs. *Par1^−/−^* mice (total of n = 45) were transplanted into irradiated (9 Gy) B6.SJL recipients along with 1×10^5^ wild type B6.SJL cells. Analysis of engraftment of competitive repopulating units (CRU) was determined by FACS analysis as the percentage of CD45.2 donor cells in the peripheral blood 4 and 16 weeks after transplantation. Mice were scored positive for CRU engraftment when the percentage of CD45.2 peripheral blood cells exceeded 0.1% and the percentage of CD45.2^+^/CD11b^+^, CD45.2^+^/B220^+^, and CD45.2^+^/CD3^+^ cells exceeded 0.02%. CRU frequencies in the blood were calculated by applying Poisson statistics to the proportion of positive recipients at different dilutions using Limiting Dilution Analyses software L-Calc (StemCell Technologies Inc.).

### Overexpression of PAR1 in murine cells

Human PAR1 cDNA was cloned into pEntry vector for gateway system (Invitrogen) and then switched from pEntry vector into the retroviral pMY-RFB destination vector, that contains a green fluorescence (GFP) expressed from an internal ribosomal entry site (IRES), by recombination reaction with LR-Clonase (Invitrogen).

Retroviral supernatants were collected as described [26]. For transduction, viruses were bound to retronectin-coated plates by centrifugation as described [28]. Lineage-depleted bone marrow cells were stimulated overnight, transduced by growth on the virus-coated plates for 24 h and sorted by FACS for EGFP-positivity. For colony assays, 1000 EGFP-positive cells per ml methylcellulose M3434 (Stem Cell Technologies) were plated. The total number of GFP-positive colonies was determined on day 10 after plating.

A total of 50,000 GFP-positive freshly transduced and FACS sorted cells were injected with 50,000 wild type bone marrow cells into the lateral tail vein of lethally irradiated (8.5 Gy) C57Bl/6N mice. Fraction of GFP-positive cells was determined by FACS in blood samples at the indicated time points after transplantation.

### Tissue array construction and immunohistochemistry analyses

Tissue array construction was performed of formalin-fixed and paraffin embedded trephine bone marrow biopsies of 152 patients diagnosed with primary, untreated AML and 7 samples of CD34^+^ cells was performed as described [29]. Informed consent was obtained from all patients and donors. For PAR1 detection, sections were incubated with the primary antibody (Thrombin R antibody (H-111), sc-5605, Santa Cruz Biotechnology Inc., Dallas, Texas, USA; dilution 1∶100). PAR1 expression was regarded as negative or positive.

### Retroviral transduction and transplantations

Retroviral transduction with MSCV2.2-MLL-AF9-IRES-GFP was performed as described [26], [28]. Briefly, bone marrow cells of wild type and Par1-knockout recipients were isolated, AKC-lysed and transduced as described previously [26]. 90.000 (MLL-AF9) GFP-positive cells were transplanted by tail-vein injection into C57Bl/6N wild type recipients, which were lethally irradiated with 8 Gy.

For secondary transplantation, bone marrow cells of leukemic mice were isolated of three independent donors of each genotype and 1×10^6^ MLL-AF9/GFP-positive cells of each donor were intravenously injected into irradiated secondary C57Bl/6N wild type mice.

Tertiary C57Bl/6N recipient mice were irradiated with 8 Gy and transplanted with 100 or 1000 ckit^+^ MLL-AF9 blasts isolated from six secondary recipients (three of each genotype). Frequencies of leukemia initiating cells (LICs) from tertiary transplanted mice were calculated using the L-Calc program (StemCell technologies, Inc.).

For the rescue experiment, leukemic spleen cells were retrovirally transduced as described above with an empty vector MSCV2.2-IRES-mCherry or with MSCV2.2-PAR1-IRES-mCherry, which contained blunt-ended human PAR1 cDNA cloned into a blunted XhoI site 5′ of the IRES. Cells were stained with a c-kit-APC antibody and sorted by FACS for c-kit, GFP and mCherry expression. 1,000 triple positive cells were transplanted into six irradiated recipient mice per group.

All transplanted mice were dosed with Cotrim (100 mg/l) (Ratiopharm, Ulm, Germany) until two weeks after transplantation. The results of the survival experiments were analysed with the log-rank non-parametric and represented as Kaplan-Meier survival curves.

### Cloning efficiency assays of murine leukemic blasts

To determine the cloning efficiency of bone marrow cells, different concentrations of bone marrow cells of untreated mice or leukemic blasts of mice that were transplanted with leukemic blasts from the primary transplantation experiment were FACS-sorted. 1, 10, 30, 100 and 300 c-kit-and, GFP-positive cells of Par1^+/+^;MLL-AF9 or Par1^−/−^;MLL-AF9 bone marrow cells were then seeded in 200 µl methylcellulose in 14 wells of a 48-well plate. 7 days later wells with one or more colonies were classified as positive. The stem cell frequency was determined by Poisson statistical analysis (L-calc software, StemCell Technologies).

## Results

### PAR1 expression profile in hematopoietic cells

**Figure 1:**
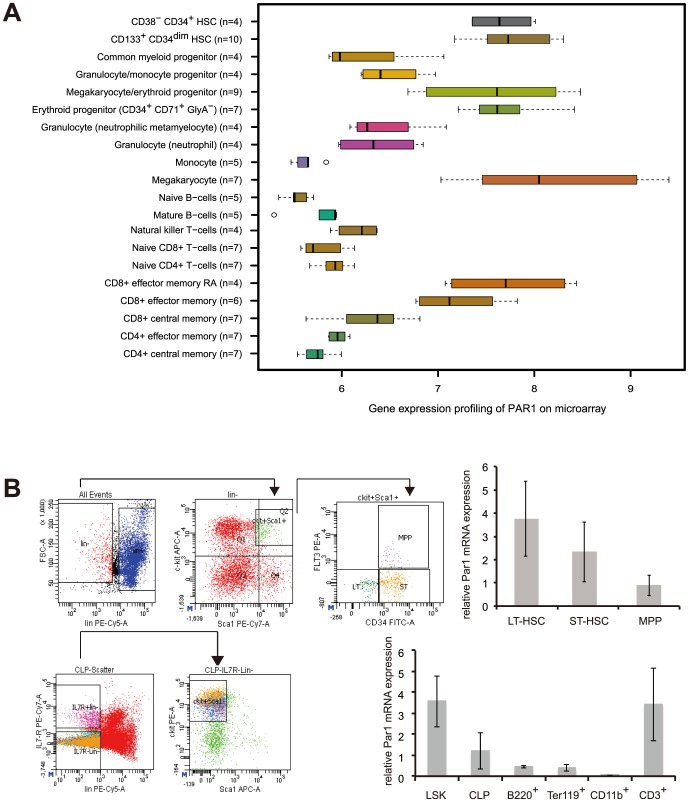
PAR1 is expressed in hematopoietic cells. **1A**. PAR1 was analyzed in mRNA microarray expression data from FACS sorted bone marrow cells [22], [23]. Highest expression was found in hematopoietic stem cells (HSC) and cells of the erythroid/megakaryocyte and of the T-cell lineage. Shown here are log arbitrary units. **1B**. Left-hand side: To sort for the different murine bone marrow subpopulation, total bone marrow was stained with lineage-markers, sca1 and c-kit. Lineage-negative, sca1^+^, c-kit^+^ (LSK) cells were further divided into long-term (LT)-HSCs as Flt3^−^CD34^−^ population, short-term (ST)-HSCs as Flt3^−^CD34^+^ cells and multipotent progenitors (MPPs) as Flt3^+^CD34^+^ cells (upper panel). Common lymphoid progenitors (CLPs) were defined as lineage-negative, IL7R^+^c-kit^+^ cells. Upper and lower right panel: *Par1* mRNA expression was determined by real-time quantitative RT-PCR using cDNA from the FACS-sorted murine bone marrow subpopulations and Par1 expression was normalized to GAPDH expression. Par1 was expressed in all hematopoietic stem/progenitor subpopulations and CD3^+^ T-cells whereas monocytes/macrophages/granulocytes (CD11b^+^) or erythrocytic (Ter119^+^) or B-cells (B220^+^) expressed low or no Par1.

Recent studies hint at a role for PAR1 in the hematopoietic system [14]. To address a potential role for PAR1 in hematopoiesis, we used published microarray data [22] to analyse PAR1 expression in multiple human hematopoietic cell types. As expected, PAR1 expression was high in cells of the erythroid/megakaryocytic lineage (Fig. 1A). Moreover, PAR1 was prominently expressed in hematopoietic stem cells (HSC), while its expression decreased upon differentiation in myeloid and lymphoid progenitor cells (Fig. 1A). Such a distinct expression pattern could not be detected for the other three proteinase-activated receptors PAR2, PAR3 or PAR4 (Fig. S1A-C).

To analyse the function of Par1 especially in the adult mice, which was not addressed yet [12], we determined Par1 expression in subpopulations of mouse bone marrow. We sorted primary cells by flow cytometry (Fig. 1B) and isolated RNA. In line with the microarray results of human hematopoietic cells (Fig. 1A), real-time RT-PCR demonstrated that Par1 mRNA was most abundant in the stem cell compartment (Fig. 1B, upper right panel). Par1 expression was also present in multipotent progenitor (MPPs, Fig. 1B, upper right panel) and common lymphoid progenitor (CLP). PAR1 was also expressed in CD3-positive T-cells in peripheral blood (Fig. 1B, lower right panel). Expression of PAR1 was notably absent in the more differentiated B220^+^, Ter119^+^ or CD11b^+^ bone marrow cells (Fig. 1B, lower right panel).

### Absence of Par1 does not interfere with normal hematopoiesis

Since Par1 was mostly expressed in stem cell fractions of primary bone marrow mouse cells, a function of Par1 in undifferentiated hematopoietic cells could be possible. We analyzed adult hematopoiesis in a previously generated *Par1*-knockout mouse model [12]. As published, we also faced a more than 50% underrepresentation of *Par1^−/−^* adult mice (see Materials and Methods).

**Figure 2:**
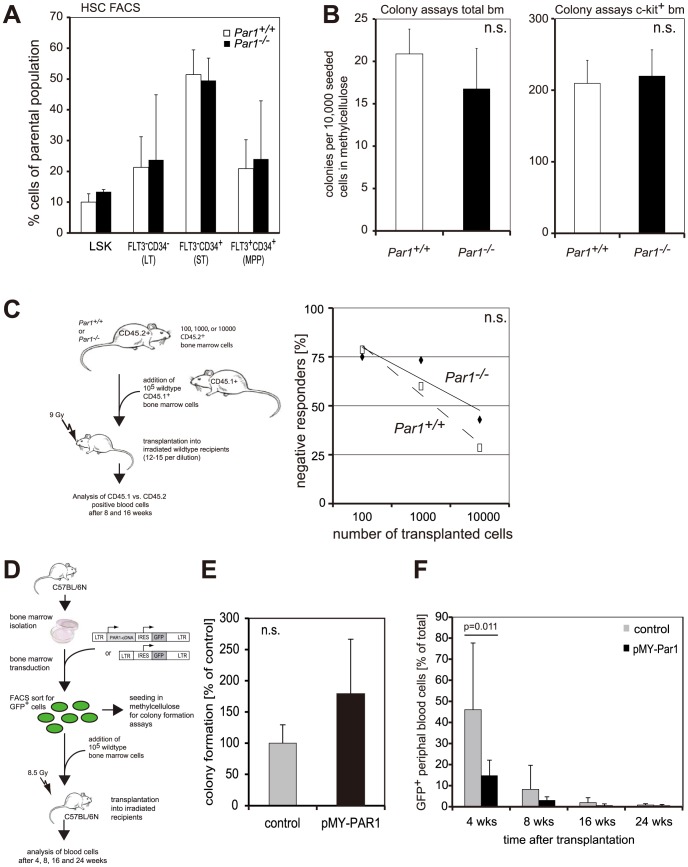
PAR1 function in proliferation and differentiation. **2A**. Stem cell FACS analysis revealed similar numbers of stem and progenitor subpopulations (n = 3 mouse pairs for each FACS). Shown here are the percentage of lin^−^sca1^+^c-kit^+^ (LSK) cells from the lineage-negative parental population and the percentage of longterm (LT)-HSCs, shortterm (ST)-HSCs and multipotent progenitors (MPP) from the parental LSK population. **2B**. Colony assays of cells from *Par1^+/+^* and *Par1^−/−^* total bone marrow (left-hand side) and c-kit+ bone marrow cells (right-hand side; n = 3 mouse pairs for each experiments). No significant changes in the ability of forming colonies were observed in any cell population. **2C**. Left-hand side: Bone marrow cells from CD45.2^+^ *Par1^−/−^* or *Par1^+/+^* mice were mixed with *Par1^+/+^* bone marrow from congenic CD45.1^+^ as depicted in the schematic overview. Right-hand side: At 16 weeks after transplantation, the number of negative responders, which were transplanted with *Par1^−/−^* bone marrow, did not differ from wild type transplanted mice. Therefore, the stem cell frequency was comparable in the bone marrow of both genotypes. **2D**. Schematic outline of the transplantation experiment using PAR1-overexpressing lineage-negative bone marrow cells compared to control cells transduced with the empty vector. **2E**. Colony formation of bone marrow cells transduced with empty vector (“control”) or with a PAR1 expressing retroviral vector (“pMY-PAR1”) was not significantly different between the two groups. **2F**. Transplantation of cells as depicted in Figure 2D lead to a significantly lower ratio of PAR1-overexpressing cells after four weeks.

We determined the function of Par1 in the regulation of stem cell growth by comparing the phenotype of wild type and *Par1*-deficient mice. We determined a spectrum of blood parameters such as white blood cells count, composition of the blood according to surface markers and hemoglobin (Table 1) and found out that the blood composition was not altered in *Par1*-deficient mice in any parameter tested. Also, the number of hematopoietic stem and progenitor cells was similar (Fig. 2A). To determine the potential of *Par1^−/−^* bone marrow cells to form colonies in methylcellulose, we performed colony assays using total bone marrow and c-kit^+^ bone marrow cells. The colony formation potential was not altered by *Par1* deficiency (Fig. 2B). Also, differentiation of these colonies was unchanged between both genotypes (data not shown). Moreover, two serial replatings of the colonies formed from *Par1^+/+^* and *Par1^−/−^* cells did not reveal differences (data not shown).

**Table 1:** Blood parameters of wild type and *Par1* ^−/−^ mice.

	3 months		6 months	
Parameters	*Par1^+/+^*	*Par1^−/−^*	*Par1^+/+^*	*Par1^−/−^*
WBC [10^3^/µl]	9.6±1.7	9.2±2.9	6.9±1.9	7.2±3.1
RBC [10^6^/µl]	8.8±0.5	9.0±0.8	8.5±0.5	8.4±1.6
HGB [g/dl]	14.0±0.9	14.2±1.1	13.3±0.6	13.1±2.4
HCT [%]	45.8±2.8	46.7±4.2	43.9±2.4	42.7±8.4
MCV [fL]	52.2±0.6	51.9±0.9	51.7±0.7	50.9±0.7
MCH [pg]	15.9±0.2	15.8±0.5	15.7±0.4	15.7±0.4
MCHC [g/dl]	30.5±0.4	30.4±0.9	30.4±0.5	30.8±1.0
PLT [10^3^/µl]	914.4±204.4	780.6±176.0	736.6±174.3	765.3±359.5
FACS				
B220^+^ [%]	54.1±3.0	55.6±5.3	50.3±6.1	42.5±11.8
CD3^+^ [%]	23.0±7.9	26.6±6.7	25.5±3.8	26.3±6.1
CD11b^+^ [%]	18.8±6.0	16.9±3.1	14.8±5.0	17.6±12.8

The data show mean values of 11 wild type and 11 *Par1^−/−^* blood analyses at the age of three months and mean values of 10 wild type and 10 *Par1^−/−^* blood preparations at the age of 6 months. WBC, white blood cell count; RBC, red blood cell count; HGB, haemoglobin; HCT, hematocrit; MCV, mean corpuscular volume; MCH, mean corpuscular haemoglobin; MCHC; mean corpuscular haemoglobin concentration; PLT, platelets. B220^+^, B-cells; CD3^+^, T-cells; CD11b^+^, myeloid cells.

Although the phenotypic number of HSCs was unchanged in *Par1*-deficient bone marrow, these cells could potentially behave differently *in vivo* and reveal a function of Par1 in hematopoietic stem/progenitor cell differentiation or proliferation after transplantation. Therefore, we transplanted wild type and *Par1*-knockout bone marrow cells in different concentrations as limiting dilution assay into wild type recipients (Fig. 2C). No significant differences were observed at 4 or 16 weeks that would indicate altered short- and long-term hematopoietic stem cell functions, respectively. *Par1*-deficient cells tended to perform better than wild type cells upon transplantation since the frequency of *Par1*-deficient cells that were detectable in the blood was higher than the frequency of wild type cells without reaching statistical significance (Fig. 2C, right-hand side).

Interestingly, bone marrow cells that retrovirally overexpressed PAR1 as depicted schematically in Fig. 2D were significantly less abundant four weeks after transplantation in wild type recipients than control cells transduced with the empty vector (Fig. 2F). These cells were not impaired in their colony formation ability (Fig. 2E). Contribution to blood cell formation was not changed (data not shown). Remarkably, Par1 did not induce a proliferative advantage in non-transformed cells.

In conclusion, neither loss nor overexpression of Par1 interferes with normal hematopoiesis.

### PAR1 expression is significantly decreased in blasts of AML patients

Thrombin receptors have long been implicated in the development of malignant diseases [16]. Especially PAR1 expression was correlated to cell migration and metastasis in different tumor entities [15], [17], [18], [19], [20], [21] but its expression and function in leukemia was unknown.

**Figure 3:**
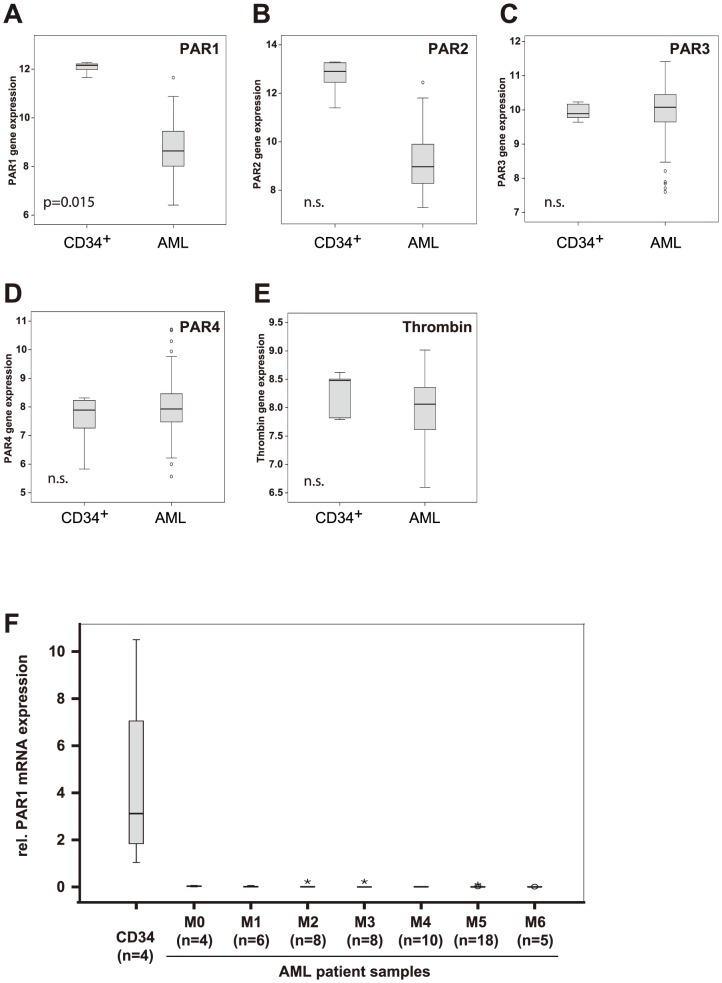
PAR1 mRNA expression in primary patient samples. PAR1 expression (**3A**) was significantly down-regulated in bone marrow cells from human Acute Myeloid Leukemia (AML; n = 67) patients compared to sorted CD34^+^ cells (n = 5) in microarray analysis, while the expression of PAR2 only showed a non-significant trend (**3B**), and the expression of PAR3 (**3C**), PAR4 (**3D**) and Thrombin (**3E**) was unchanged. Shown here are log arbitrary units. **3E**. PAR1 expression was significantly downregulated in bone marrow cells from human Acute Myeloid Leukemia (AML) patients compared to CD34-positive bone marrow cells. PAR1 expression was determined by qRT-PCR and normalized to GAPDH expression level.

**Figure 4:**
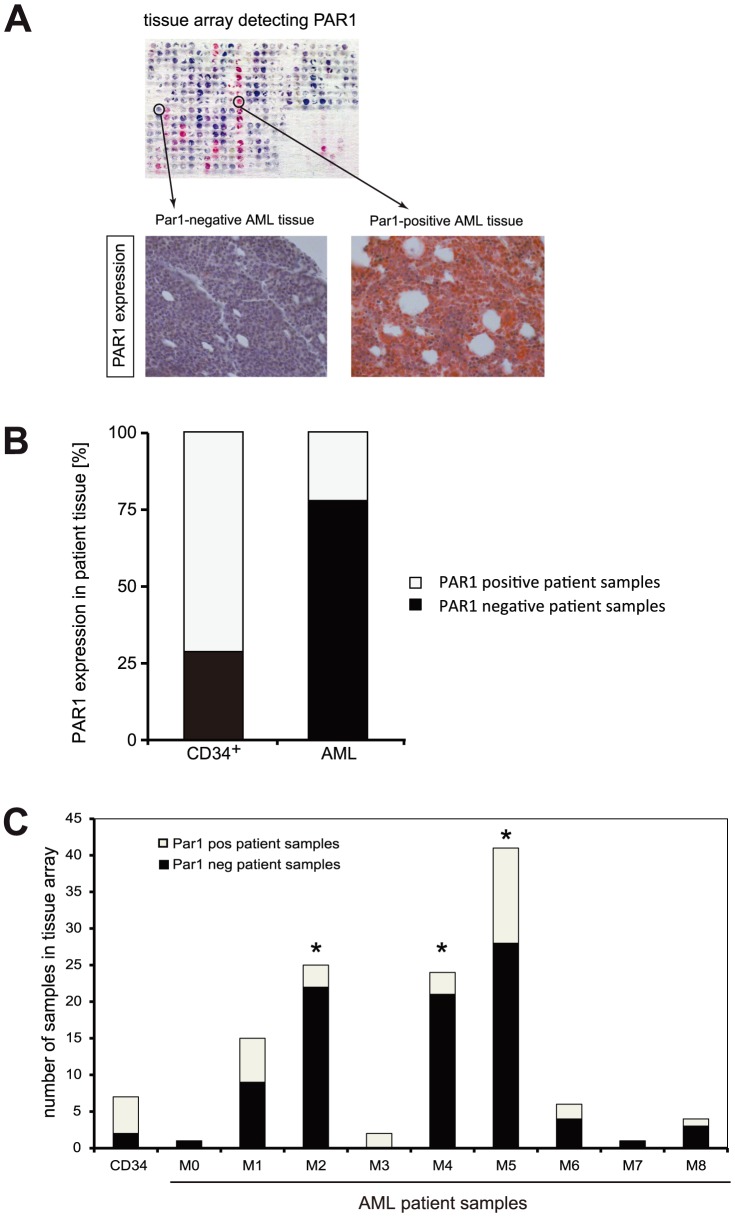
PAR1 expression in primary patient samples. **4A**. Micrographs of Tissue Array analysis from NBM and AML patients stained with anti-PAR1 antibody and Fast-Red secondary antibody contrasted with hematoxylin and eosin. Overview (upper panel) and magnification of one example of CD34^+^ and AML samples that were defined PAR1-negative (lower left) and PAR1-positive (lower right). **4B**. Quantitative Tissue Array analysis of PAR1 expression using categories of staining intensity as positive or negative. Significantly more AML patient samples were negative for PAR1 expression than CD34^+^ healthy patient samples (p = 0.003, Chi-square test). **4C**. PAR1 protein was significantly less abundant in bone marrow cells from human Acute Myeloid Leukemia (AML) patients compared to CD34-positive bone marrow cells in Tissue Array samples. *p<0.05, Chi-square test.

Although the activity of receptors is tightly regulated on protein levels, PAR1 recovery might also rely on new protein synthesis and therefore on the abundance of its mRNA in some cell types including cells from the hematopoietic system [30]. Hence, we analyzed the expression of PAR1 in a large set of leukemia patient samples using Gene expression microarrays for mRNA analyses (Fig. 3) and real-time RT-PCR (Fig. 3) and a tissue microarray for protein expression (Fig. 4).

The mRNA analysis of five CD34^+^ cells of healthy donors and 64 AML patients revealed that PAR1 expression was markedly lower in AML blasts than in CD34^+^ progenitor cells (Fig. 3A), whereas the expression of the other three family members did not differ (Fig. 3B-D). Detailed analysis of PAR1 expression demonstrated its significant downregulation in all FAB subtypes of AML (Fig. S2A). AML patients with high PAR1 expression (level >9 log arbitrary units in this microarray analysis) did not reveal changes in hemoglobin, LDH, number of platelets, white blood cells or blasts in the blood or bone marrow at the time of diagnosis compared to patients with lower PAR1 expression (level <9 log arbitrary units in this microarray; data not shown). PAR1 expression also did not influence overall survival or relapse-free survival (data not shown). Of note, the expression of the main upstream regulator of PAR1 function, the ligand Thrombin, was unchanged (Fig. 3E).

We confirmed PAR1 expression by quantitative real-time RT-PCR in CD34-positive cells from healthy patients and samples from AML patients (Fig. 3F) in an independent cohort of patients. Compared to CD34^+^ cells, PAR1 expression was again significantly decreased in all AML subtypes (Fig. 3F).

To analyze the protein expression of PAR1 in control and AML patient samples, we used immunohistochemical detection of PAR1 on a tissue array that included CD34-positive cells as well as sections of bone marrow punches. Remarkably, tissue array analysis of PAR1 expression revealed that PAR1 was more prominently expressed in CD34^+^ cells from healthy volunteers compared to AML blasts (Fig. 4A and B). Only 30 out of 119 AML patient samples showed PAR1-expression (25%), whereas 5 out of 7 samples of CD34^+^ cells were positive for PAR1-expression (71%; Fig. 4B) (p = 0.008, Chi-square test [31]). The finding of PAR1 protein expressing AML samples (Fig. 3F) suggest that PAR1 protein might be present although mRNA levels were very low in most AML patients. Immunohistochemistry staining might also pick up other PAR proteins, which might be expressed in certain AML samples (Fig. 3C and D). Nonetheless, PAR1 mRNA and protein data are highly concordant with loss of expression in most of the specimens. Also, these results were in accordance with the observed differences in the Par1 expression in sorted mouse bone marrow cells, in which Par1 was highly expressed in the stem cell compartment and in progenitor cells (Fig. 1B). In line with the results obtained in the microarray analysis, PAR1 expression did not correlate with hemoglobin, number of platelets, white blood cells or blasts in the blood or the bone marrow at the time of diagnosis (data not shown). Also, different PAR1 levels were not associated with the overall survival time or the relapse-free survival of the patients (data not shown). Interestingly, in this analysis PAR1 expression was especially low in AML M2, M4 and M5 (Fig. 4C).

### PAR1-deficiency enhances leukemic stem cell potential

The observation that PAR1 expression differed significantly in human acute myeloid leukemia and especially in AML M4 and M5 (Fig. 4C) led us to analyze Par1 functions in murine leukemogenesis. To model AML *in vivo*, wild type or *Par1*-knockout (−/−) bone marrow cells were retrovirally transduced with the leukemogenic MLL-AF9, which occurs in human AML M5 [32] and reliably induces an AML in mice [33], [34], [35].

**Figure 5:**
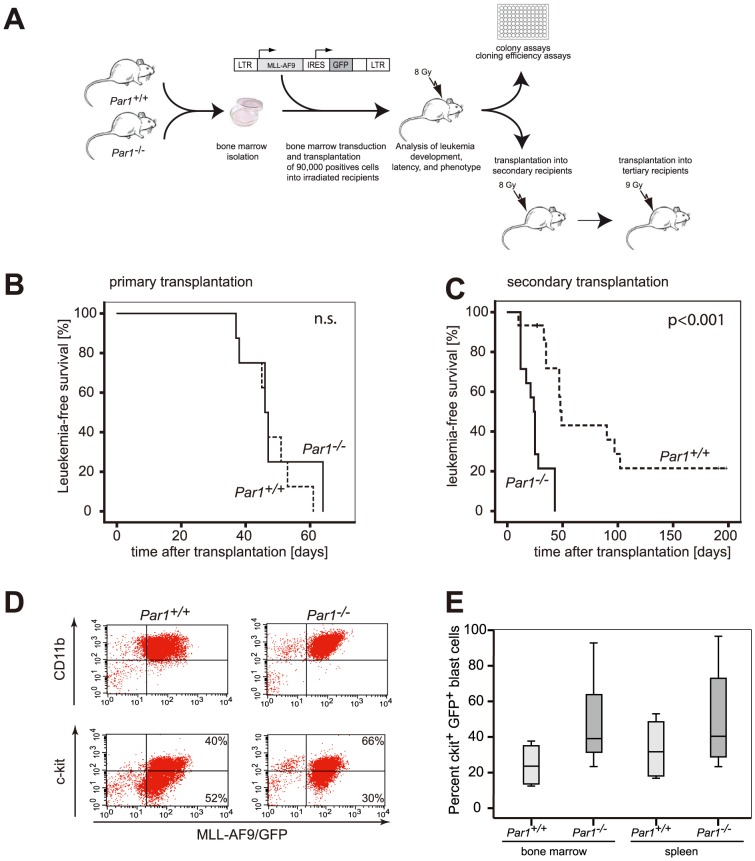
Absence of *Par1* accelerates MLL-AF9 driven murine leukemogenesis. **5A**. Schematic overview about the performed transduction and transplantation experiments. Bone marrow isolated from *Par1^+/+^* or *Par1^−/−^* mice was retrovirally transduced with MLL-AF9/GFP. Equal numbers of positive cells were transplanted into lethally irradiated recipients, which were then subjected to different analyses and subsequent serial transplantations. **5B**. Survival curves of recipient mice which were transplanted with bone marrow cells of *Par1^+/+^* or *Par1^−/−^* mice that were retrovirally transduced with MLL-AF9 (n = 8 of each genotype). Cells of both genotypes led to a fatal leukemic disease with comparable latency. **5C**. Survival curves of secondary recipient mice which were transplanted with bone marrow cells of leukemic mice derived from the primary transplantation shown in Fig. 5B. The secondary recipients of *Par1^−/−^*;MLL-AF9 cells (n = 14) died after a significantly shorter latency than mice transplanted with *Par1^+/+^*;MLL-AF9 primary blasts (n = 15; p<0.001). **5D**. The phenotypic analysis of blasts of the secondary leukemic mice did not reveal differences in CD11b expression between *Par1^+/+^*;MLL-AF9 and *Par1^−/−^*;MLL-AF9 cells. **5E**. *Par1^−/−^*;MLL-AF9 transplanted mice (n = 8) exhibited a strong tendency towards higher percentages ofc-kit expressing cells in spleens (p = 0.055, t-test) and bone marrow (p = 0.22, t-test)compared to *Par1^+/+^*;MLL-AF9 transplanted mice (n = 4).

Transplantation of 90.000 positive cells as assessed by GFP expression (Fig. 5A) of bone marrow cells retrovirally transduced with the oncogene MLL-AF9 induced myeloid leukemia both in wild type and *Par1^−/−^* bone marrow cells with comparable latency, penetrance, and morphology (Fig. 5B and data not shown). Acute myeloid leukemia in mouse models is defined by transplantability into secondary recipients [36]. Transplantation into secondary recipients assesses leukemic stem cell function. Interestingly, *Par1-*deficiency significantly accelerated the leukemic disease in secondary recipients (Fig. 5C; p<0.001). Of note, this finding was cell intrinsic, since all recipients were of *Par1* wild type genotype. Both genotypes generated an acute myeloid leukemia after secondary transplantation (Fig. 5D).

In murine MLL-AF9 leukemias, the c-kit positive fraction contains the leukemic stem cells [33]. We determined the fraction of c-kit^+^ blasts within the GFP^+^ cells to determine whether the phenotypic stem cell fraction was altered. In spleen as well as in bone marrow, the fraction of c-kit^+^ stem cells was increased in the *Par1^−/−^* blasts (Fig. 5E). In spleen, the mean percentage of c-kit positive cells was 24.3% in wild type leukemias but 48.1% in leukemias with *Par1*-deficiency. Also, half of the leukemias with wild type Par1 showed less than 20% c-kit positive cells whereas all *Par1*-deficient leukemias harbored more than 20% of c-kit positive cells (Fig. 5E).

### Par1 restricts the leukemic stem cell pool size and function

**Figure 6:**
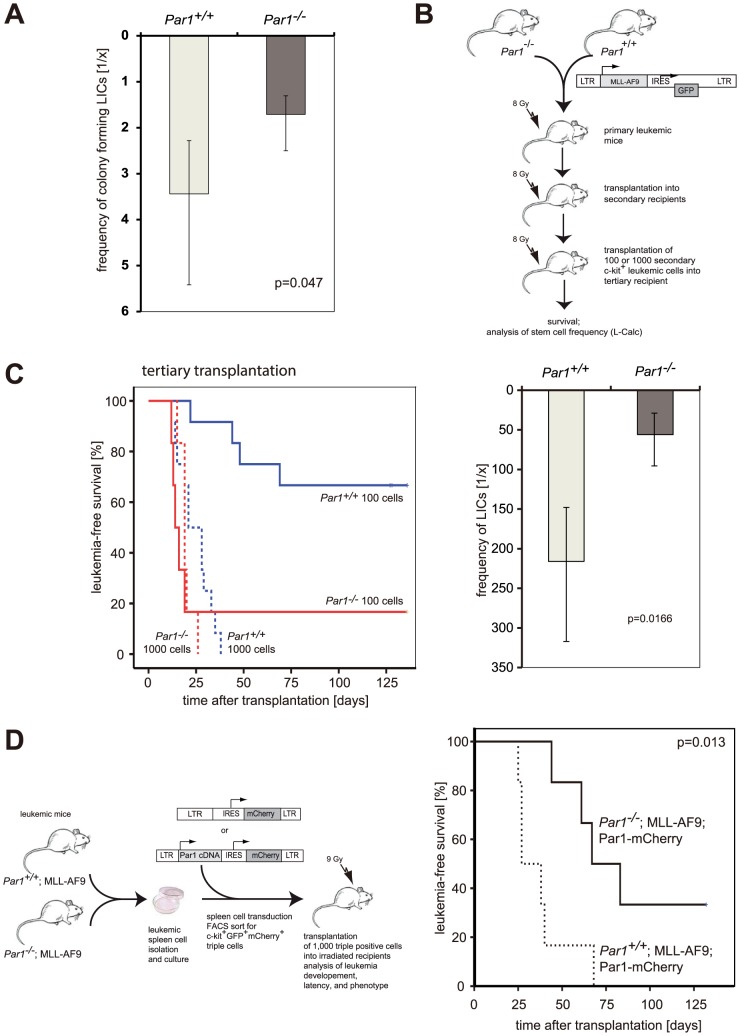
Leukemia initiating cells are regulated by Par1. **6A**. For a cloning efficiency assay, *Par1^+/+^*;MLL-AF9 or *Par1^−/−^*;MLL-AF9 bone marrow cells from leukemia-transplanted mice were FACS-sorted and 1 to 300 c-kit^+^GFP^+^ cells were seeded in semi-solid medium in a 48-well plate. *Par1^+/+^* cells had a clone forming frequency of 1/3.4, while the frequency was much higher in *Par1^−/−^* cells (1/1.7; p = 0.047). Shown here are the mean results of three independent experiments. **6B**. Schematic overview about the serial transplantations performed with MLL-AF9 leukemic blasts. **6C**. Left-hand side: Kaplan-Meier plot illustrates the leukemia-free survival of tertiary transplanted mice. After tertiary transplantation, transplantation of 100 MLL-AF9 c-kit^+^ leukemic blasts revealed a significant elongated life span of mice transplanted with *Par1^+/+^* cells (n = 12) compared to *Par1^−/−^* cells (n = 6; p = 0.002). Transplantation of 1000 cells did not reveal significant difference concerning the overall survival. Right-hand side: The frequency of leukemia-initiating cell was calculated according to the results shown in the left-hand plot by using the program L-Calc. *Par1^+/+^* leukemia-initiating cells appeared with a frequency of 1/216, while the frequency was much higher in *Par1^−/−^* cells (1/56; p = 0.0166). **6D**. Overexpression of PAR1 in *Par1^−/−^* MLL-AF9 leukemic spleen cells extents the life time of transplanted mice. Left-hand side: Schematic outline of the experimental of the transplantation. Right-hand side: Kaplan-Meier plot reveals the significant longer latency of leukemia in mice transplanted with *Par1*-deficient compared to wild type MLL-AF9 c-kit^+^ blasts overexpressing PAR1.

We hypothesized that loss of *Par1* led to an expansion of the leukemic stem cell pool with enhanced stem cell activity. To test this hypothesis, we performed cloning efficiency experiments of c-kit^+^GFP^+^ bone marrow cells (as depicted in Fig. 5A) from leukemic mice after secondary transplantation to determine the fraction of MLL-AF9 expressing cells that could give rise to clonal growth. MLL-AF9-positive cells from secondary transplanted mice were sorted according to their c-kit- and GFP-positivity and seeded in cell numbers from 1 to 300 cells per well in methylcellulose in 48-well plates and the clone forming efficiency was determined according to Poisson-statistics. *Par1^−/−^*;MLL-AF9 cells exhibited a cloning efficiency of 1/1.7, while the cloning efficiency of *Par1^+/+^*;MLL-AF9 cells (1/3.4) was two times lower (Fig. 6A; p = 0.047). Interestingly, non-transduced c-kit^+^ *Par1^−/−^* bone marrow cells, which were seeded in the same way to determine their cloning efficiency capacity, did not form more clones than wild type bone marrow cells (data not shown).

To determine the frequency of leukemia initiating cells (LICs) *in vivo*, we transplanted 100 and 1000 c-kit^+^ cells of secondary transplanted leukemic mice serially into irradiated tertiary recipients as depicted in Fig. 6B. Recipients that received *Par1^+/+^* blasts survived significantly longer than those that received *Par1^−/−^* cells (Fig. 6C, left-hand side). From this transplantation, we determined the frequency of LICs in both genotypes according to the positive responder mice that died due to leukemia by Poisson-statistics (Fig. 6C, right-hand side). The frequency of LICs was about four times higher in absence of *Par1* (1/56) than in presence of *Par1* (1/256; p = 0.0166), which most likely contributed to the shortened latency and higher penetrance in this transplantation.

### Re-expression of Par1 restricts leukemic stem cell function

Since the absence of Par1 enhanced leukemogenesis, we hypothesized that re-introduction of Par1 expression in *Par1*-deficient leukemic blasts could decelerate the disease. Hence, we used MLL-AF9 positive splenic wild type and *Par1^−/−^* blasts from primary transplanted mice (Fig. 5B) and transduced them with a retroviral construct that expressed human PAR1 and the red fluorescent protein mCherry or as a control the empty vector only expressing mCherry. Cells were sorted by flow cytometry for their expression of MLL-AF9 (GFP), mCherry and c-kit as a marker for MLL-AF9 LICs. Each mouse received 1,000 triple-positive cells (Fig. 6D, left-hand side; n = 6 for each group). As expected from the results obtained from transplantation of 1,000 c-kit^+^ MLL-AF9 splenic cells before (Fig. 6C), blasts of both genotypes transduced with the control vector led to a rapid disease with comparable latency (Fig. S3). In contrast, overexpression of PAR1 in *Par1^−/−^* blasts significantly extended the survival time of recipient mice compared to mice transplanted with PAR1-overexpressing wild type MLL-AF9 blasts (Fig. 6D, right-hand side; p = 0.013). Moreover, overexpressing of PAR1 in cells with wild type levels of endogenous Par1 do not exhibit a significantly altered survival time compared to the control groups (Fig. S3).

In conclusion, Par1 acts as controller of leukemic stem cells in MLL-AF9 triggered murine leukemia and leukemic mice lacking *Par1*-expression in their blasts benefit from recovery of Par1 function.

## Discussion

Our study reveals that PAR1 is especially expressed in healthy hematopoietic stem cells, whereas PAR1 expression is markedly lost in acute myeloid leukemia. The loss of Par1 leads to enhanced leukemic stem cell function *in vivo*.

Members of the hematopoietic serine protease superfamily that activate PARs, such as cathepsin G, neutrophile elastase and proteinase 3, may play an important role in myeloid biology [37]. Patients, who suffer from hematological disorder or congenital neutropenia frequently exhibit mutations in genes for neutrophil serine-proteases or show alterations in its expression, localisation or activity [38]. Nonetheless, PAR1 is not required in normal hematopoiesis and HSC function. The dispensability of Par1 in these processes might rely on redundant action of other proteinase-activated receptors, as it was already assumed for Par2 in thrombin-induced responses in *Par1^−/−^* platelets [12]. Moreover, persistent thrombin signalling in *Par3*-deficient platelets led to the identification of Par4 [39]. To determine the role of other PAR family members in hematopoiesis will require further experiments like the generation of Par1/Par2-double deficient mice, which might be difficult using the straight knockout mice due to the limited survival of both single-mutant mouse models [12], [40]. For this kind of experiments, the generation of conditional knockout mouse lines might be necessary.

Up to now, PAR1 was assigned to oncogenic function in many tumor entities [2], [16], [18], [19], [41]. We were intrigued by the widespread loss of PAR1 in AML blasts by integrating the expression levels of PAR1 in three different leukemia patient cohorts on mRNA and protein levels. We therefore tested the role of *Par1*-deficiency in mouse leukemia. The oncogenic translocation product MLL-AF9 is frequently found in human leukemias [42], [43]. We took advantage of the fact that PAR1 protein expression was downregulated in human AML patient samples of FAB subtypes M4 and M5 in our tissue arrays (Fig. 5C) and that these AML subtypes can be modelled by the retroviral introduction of MLL-AF9 in hematopoietic progenitors [34]. The AML-like phenotype is readily induced by MLL-AF9 in mice, either as a stable knockin [44] or by transient retroviral transduction and transplantation [34]. The widely-accepted concept of leukemic stem cells [45] can be recapitulated very consistently in this leukemia model, since predominantly the c-kit^+^ fraction of MLL-AF9 positive leukemic blasts is transplantable and capable of self-renewal comparable to normal HSCs [34], [35].

We discovered that Par1 expression restricted the pool of functional leukemic stem cells, rather than promoting it as an oncogene. Many receptors have been assigned as oncogenes, also in leukemogenesis. Prominent examples are the receptor-tyrosine kinases like FLT3 [46] and c-KIT [47]. But usually, these receptors are overexpressed or constitutively active due to mutations, which lead to overactivation of downstream targets, or to misactivation of other targets. In the case of PAR1, the mechanism of action in leukemogenesis might be different. Absence of Par1 enhances leukemia development, which might indicate *vice versa* that wild type expression of Par is able to suppress leukemogenesis to a certain extent. Recently, it was shown that Par1 signal transduction might occur via the RhoA/ROCK1 pathway [14], [48], which is also implicated to influence hematopoietic stem cells [49]. It will be interesting to investigate to which extent an alteration in this or another signal pathway in involved in the phenotype of *Par1*-deficient MLL-AF9 leukemic mice.

Although it was somewhat surprising that Par1 acted as a suppressor of stem cell function in leukemia, whereas it is implicated as an oncogene in other cancer entities, several other prominent factors also display such divergent functions. One example is the polycomb complex protein EZH2 that acts as an oncogene i.e. in prostate and breast cancer [50], [51], while it suppresses T-cell leukemia development in mice [52]. In addition, Notch1 signalling is intensively studied and discussed as oncogene in different tumors and as tumor suppressor in leukemias [53], [54], [55]. Therefore, it is quite possible that Par1 acts with divergent outcome in different cancers. In addition, also its close relative Par2 was already identified as tumor suppressor in a model for skin carcinogenesis [56], although Par2 was also mostly accepted as oncogene [57], [58], which illustrates the diverse functions that can be expected in this receptor family.

Finally, the fact that mice transplanted with *Par1*-deficient MLL-AF9 blasts benefit from the re-activation of Par1-expression might suggest that this could also help as a therapy for patients initially expressing very low or no PAR1. Rendering leukemic stem cells responsive to leukemia therapy is still a big task with the goal to be able to ultimately eradicate the disease (reviewed in [59]). Further studies on the role of Par1 in different leukemias might help to understand leukemic stem cell function and to develop molecular therapies to target these cells.

## Supporting Information

Figure S1 **PAR2, PAR3 and PAR4 expression in hematopoietic cells.** Expression of PAR2 (S1A), PAR3 (S1B) and PAR4 (S1C) was analyzed in published microarray data from FACS sorted bone marrow cells (22, 23). None of them was prominently expressed in hematopoietic stem cells (HSC). Shown here are log arbitrary units.
(EPS)

Figure S2 **PAR1 expression determined in microarray analysis according to FAB subtypes.** PAR1 is significantly less expressed in all FAB subtypes tested by microarray analysis.
(EPS)

Figure S3 **Empty vector controls to PAR1-overexpression transplantation.** Survival curve of mice that were transplanted with MLL-AF9-induced Par1-wt and -ko leukemic spleen blasts that were additionally transduced with MSCV-IRES-mCherry empty vector. Mice that were transplanted with these cells exhibited leukemia initiation with comparable latency.
(EPS)
